# Migrants and emerging public health issues in a globalized world: threats, risks and challenges, an evidence-based framework

**DOI:** 10.3134/ehtj.09.010

**Published:** 2010-03-31

**Authors:** BD Gushulak, J Weekers, DW MacPherson

**Affiliations:** 1 Research Section, Migration Health Consultants, Ontario, Canada; 2 Migration Health Department, International Organization for Migration, Geneva, Switzerland; 3 Faculty of Health Sciences, McMaster University, Ontario, Canada

## Abstract

International population mobility is an underlying factor in the emergence of public health threats and risks that must be managed globally. These risks are often related, but not limited, to transmissible pathogens. Mobile populations can link zones of disease emergence to lowprevalence or nonendemic areas through rapid or high-volume international movements, or both. Against this background of human movement, other global processes such as economics, trade, transportation, environment and climate change, as well as civil security influence the health impacts of disease emergence. Concurrently, global information systems, together with regulatory frameworks for disease surveillance and reporting, affect organizational and public awareness of events of potential public health significance. International regulations directed at disease mitigation and control have not kept pace with the growing challenges associated with the volume, speed, diversity, and disparity of modern patterns of human movement. The thesis that human population mobility is itself a major determinant of global public health is supported in this article by review of the published literature from the perspective of determinants of health (such as genetics/biology, behavior, environment, and socioeconomics), population-based disease prevalence differences, existing national and international health policies and regulations, as well as inter-regional shifts in population demographics and health outcomes. This paper highlights some of the emerging threats and risks to public health, identifies gaps in existing frameworks to manage health issues associated with migration, and suggests changes in approach to population mobility, globalization, and public health. The proposed integrated approach includes a broad spectrum of stakeholders ranging from individual health-care providers to policy makers and international organizations that are primarily involved in global health management, or are influenced by global health events.

## Introduction

Several current emerging threats and risks exposing public health vulnerabilities are linked to global processes, such as economics, trade, transportation, environment and climate change, and civil security. Many of these processes are influenced or affected by the migration and mobility of human populations.^1^, ^2^ International migration, which is a supporting component and a consequence of globalization,increasingly affects health in migrant source, transit, and recipient nations.^3^ The flow of populations between locations with widely different health determinants and outcomes creates situations in which locally defined public health threats and risks assume international or global relevance. This fact is illustrated by the rapid awareness,detection, and response to the emergence of a novel influenza A/H1N1 virus in the spring of 2009.^4^, ^5^ Global demographic predictions indicate that the forces promoting and supporting international migration will continue to do so, and may become stronger in all regions of the world as populations attempt to move up gradients of opportunity (such as economic, educational, security, health).^6^

Both the diversity (The term ‘diversity’ describes the dissimilarities between host and migrant populations relevant to the determinants of health (such as genetics and biology, environment, behavior, and socioeconomics);‘disparity’ reflects the burden of inequalities present at both individual and societal levels, which affects the determinants of health.) and the nature of modern mobility and migration frequently exceed the capacities of and thereby challenge existing policies and programs designed to address migration and health. The goals of this paper are to promote a change in thinking and approach to global health issues that reflects emerging evidence and importance of population-based factors, as opposed to disease or pathogen-based regulatory frameworks that have been traditionally used. This approach focuses on factors of human population mobility and shifting demographics that affect both health determinants and disease outcomes.

Attention in the public health community is traditionally drawn toward the potential and real effects of infectious diseases associated with migration.^7^ Common examples observed in nations that receive migrant populations include tuberculosis^8^–^11^ and hepatitis B.^12^–^14^ However, sustained migration between disparate health environments also affects the longer-term epidemiology of chronic noninfectious diseases; this has a downstream impact on health promotion, health services for disease prevention and management, and occupational health outcomes at migrant destinations. As a result of these impacts, migration exerts increasing influence on public health policy,^15^ education of health-service providers,^16^ health system design, and service delivery.^17^ Examples of these influences include the need to regulate or manage the use of alternative medicines and pharmaceuticals imported by migrants, or the introduction of alternative medical procedures.^18^–^20^

In terms of public health, migration has implications for recognition of threats, as well as for surveillance and response capacity.^21^ Migration also influences broader aspects of the ‘health of the public,’ including the background burden of chronic or latent diseases (both infectious and noninfectious) and patterns of preexisting immunity; it also influences the use and uptake of disease prevention and health promotion interventions, and health-care service utilization in general.^22^, ^23^ Ensuring that necessary information is both available and understood by diverse communities is an increasingly important aspect of public health planning and preparedness^24^–^26^ in nations with large mobile populations. This was recently shown by responses to the threat of influenza A/H1N1 importation, which included quarantine, isolation, or preventive interventions.^27^–^29^

Traditional regulatory approaches to public health risks in the context of migration are commonly disease oriented^30^ or event based.^31^ Frequently, these concepts assume or are based on the view that a degree of homogeneity or similarity characterizes migrant populations; they also relate to the administrative or legal status of the population. This article puts forth the observation that many of the important public health aspects of migration originate from or are based on the diversity and disparity of the populations themselves and extend beyond the legal and temporal processes involved in changing one's residence. Addressing migration-associated health threats and risks will be more effectively accomplished if approached from this population-based framework, rather than from traditional diseaseor immigration status-based views. It is proposed that the development of standardized, programmatic approaches to health and migration that are based on collective international evidence would be an effective strategic and operational approach to global health.

Failure or further delays in addressing emerging health issues associated with migration and population mobility will continue to impair the effectiveness of policies and programs designed to tackle modern global health challenges.^32^, ^33^

## Methods

The peer-reviewed literature was accessed through electronic searchable sites (PubMed, ProMED, and others) using standard search strategies for literature related to migrants, mobile populations, and specific disease outcomes. In addition, publicly available reports from international and national organizations and agencies were accessed for information on migrants, mobile populations, and health. Organizations and agencies included The World Health Organization, International Organization for Migration, International Labour Organization, other United Nations (UN) agencies, World Bank, Centers for Disease Control and Prevention (Atlanta, USA), European Centres for Disease Prevention and Control, The Health Protection Agency (UK), and others. In all the literature or reports, population demographics, source country, reporting region, and health outcome measurements were sought. As the study involved no contact with patients or individuals or personal medical information, research ethics approval was not sought.

## Human population mobility

Several factors associated with human population mobility make it a significant determinant of future health threats and risks for all regions of the world. These include the number of people on the move, as well as the diversity and disparity of population characteristics between source, transit, and recipient destinations. Taken together, these factors exert a significant influence on the globalization of disease threats and risks. A third factor that continues to challenge objective measurement of health outcomes in migrants is the lack of standardization in terminology that applies to mobile populations. Doing so would allow correlation and differentiation of populations on the basis of health-risk characteristics rather than administrative categories.

### The demographics of modern migration

Part of the growth in migration is simply due to the increase in human population (the rate of growth is no longer increasing). The global population in 1950 was estimated at ~2.6 billion people. The global population estimate in 2008 was 6.7 billion.^34^ In 1960, it was estimated that there were 76 million international migrants.^35^ Current estimates of international migrants place their numbers at ~200 million. If this population were considered a national population,international migrants would be the world's fifth largest nation.

A 2006 report from the UN^36^ showed that 75% of all migrants resided in 30 nations; migrants constitute at least 20% of the total population of 41 nations; 60% of international migrants live in high-income economies;30% of migrants have moved from a low-to-middle income country to a similar economically designated nation; and 30% of migrants have moved from a low-to-middle income nation to a high-income nation. (‘south-to-south’ migrants are about as numerous as ‘south-to-north’ migrants.) Of the ~200 million international migrants reported by the UN in 2005, ~20% arrived in the United States alone. These trends can be associated with profound demographic impacts on migrant source and destination countries.

These global population figures, although important in their magnitude, also reflect significant differences in the demographic and health determinants of the migrants themselves. There are millions of migrant workers who leave their families behind to be supported by their financial remittances. The migrant worker population is increasingly composed of women, particularly within Asia. These population figures also comprise refugees and internally displaced individuals who seek a safe haven and security after fleeing conflict and disaster (see Table 1).

**Table 1:** Global estimates of migrant populations

*Category of migrant*	*Population estimates*
Regular immigrants	Annual flow of ~2.4 million (2005) with a stock of ~200 million^127^
International students	~2.1 Million (stock in 2003)^128^
Migrant workers	~86 Million (stock in 2005)^129^
Refugees	16 Million (stock in 2007) Source United Nations High Commission for Refugees^125^
Asylum seekers or refugee claimants	650,000 (stock in 2007)^125^
Temporary— recreational or business travel	900 Million per year (2007)^126^
Trafficked (across international borders)	Estimated 800,000 per year (2006)^130^
Internally displaced	51 Million (stock in 2007) includes those displaced by natural disasters and conflict^125^

In addition, there are those populations for whom the numbers and statistics are less definitive. This refers to population movements of migrants without legal permission or authority to migrate, who enter and reside unofficially in a host country. The clandestine nature of these unauthorized migrant (‘Unauthorized migrants’ is emerging as the preferred terminology for international migrants who arrive without the necessary approvals, permissions, or documents (such as identification, citizenship cards, visas, passports, and other travel documents). Irregular migrants, undocumented migrants, and those who are smuggled or trafficked outside their own country are included in this terminology.) flows, including smuggling and trafficking, makes the determination of their numbers difficult and estimates inexact. The quality of data for unauthorized migrants decreases as the migration process becomes less formally organized.

### Nonstandardized use of terminology describing migrants

A major challenge in assessing health impacts of migration is the lack of agreed definitions and consistency in the use of terminology to describe migrant populations over time. Some migratory movements may involve international travel across formal boundaries, whereas others, such as rural–urban^37^ migration and the internal displacement of populations through conflict or disaster may remain internal national processes.^38^ Each of the processes and movements can be associated with several descriptive or definitive terms that can vary over time, use, location, and legal or administrative standards. The terminology used to describe a group of migrants in one location, for example, immigrants, may refer to a different migrant group in another setting. In some settings, the term ‘immigrant’ refers only to those accepted as residents in the destination country and granted legal status to remain there. In other jurisdictions, the term ‘immigrant’ may be used to refer to any foreign national including those without a formal legal status.

Although many health risks are associated with movement between locations with different health determinants and outcomes, many definitions of migrant populations currently are based on administrative measures, such as the duration of stay. The UN distinguishes migrants according to the duration of stay, classifying them into long-term migrants (residence in a country other than the usual place of residence for >12 months) and short-term migrants (a period of residence of >3 months but <1 year). This definition does not apply to those individuals traveling for business, to visit friends or relatives, to seek medical treatment, or to undertake pilgrimage.^39^ This variability in the use of terminology and lack of direct descriptions related to health creates challenges in the analysis and interpretation of health outcomes associated with migration. International organizations and agencies are attempting to standardize the terminology of migration, but these activities are largely based on administrative and residency principles and may not adequately reflect the health characteristics and determinants of the person or population.^40^

Furthermore, many modern migration flows are temporary or reciprocal, reflecting the global integration of economic, educational, and labor markets. Such movements include populations composed of temporary and seasonal workers, international students, or medical tourists, as well as the growing numbers of those with dual or multiple citizenship and right of residence. These populations, although experiencing and reflecting the health and public health influences of migration, are not systematically captured in traditional national or international immigration statistics. It has been proposed that the health aspects of migration can be better addressed through a standardized, population health-based functional approach rather than by the administrative consideration of processes of modern migration (see Table 2; modified from Gushulak and MacPherson^41^).

**Table 2:** Health influences related to phase of mobility (modified from Gushulak and MacPherson^41^)

*Phase*	*Examples*	*Outcomes/risks*
Existing pre-departure influences	Endemic diseases	Departure/arrival with health indicators of origin:
	Level of development	Differing incidence and prevalence of illness
	Access to care	Differences in awareness of and use of health-care services:
	Availability of care	Preventive
	Differences in linguistic and cultural background as compared with destination	Promotional
		Diagnostic
	Possible forced or nonvoluntary departure from emergency situations	Therapeutic
Influences during travel	Trauma (physical and mental)	Greater prevalence of illness resulting from torture, trauma, abuse, and climatic exposure
	Deprivation	
	Violence	Refugees
	Climatic exposure	Refugee claimants or asylum seekers
	Injury	Unauthorized migrants, including trafficked/smuggled migrants
Post-arrival influences	Administrative/legal restrictions on access to services or care	Awareness of and access to health services by migrants may be limited by immigration status, poverty, language, culture, and discrimination
	Poverty	
	Language and cultural isolation	Working conditions may be associated with health risks,
	Occupational risks	exploitation, and abuse:
	Duration of stay at destination	Migrant agricultural laborers
		Commercial sex workers
		Illegal workers
		Trafficked migrants
Health influences associated with return travel	Changed health environment at origin (health systems improvements or declines)	Introduction of disease, acquired abroad, into home country Populations making return journeys to the place of origin (particularly children born at new destination) may be at increased risk of disease or illness:
	Children of foreign-born parents may have no exposure to risks present at origin	Visiting friends and relative travelers
		Locally born children of foreign-born parents
		Risks may return to new home after visit or may be introduced during travel

### Emerging health threats, risks, and challenges resulting from migration

Identifying threats related to migrant populations has been driven by historical outbreaks of transmissible infectious diseases of public health significance, such as plague and cholera. As seen in recent years with the severe acute respiratory syndrome (SARS, 2003) and influenza A/H1N1 (2009 pandemic), many national responses with regard to migrant populations tend to be traditional, on the basis of the principles of border inspection, isolation or quarantine, and exclusion.

In migration health, threat and risk identification, assessment and management rarely occur ‘pre-event.’ Examples of poorly studied health threats of potential societal and public health importance include domestic violence against migrant women in destination locations,^42^, ^43^ long-term impact of dietary changes^44^, ^45^ on the incidence of cardiovascular disease,^46^ diabetes,^47^ and certain forms of cancer in foreignborn migrants and their locally born offspring,^48^ or the importation of health services or pharmaceutical products^49^ from less-regulated environments, representing traditional but often unregulated or unmonitored patterns of self-care.^50^

Efforts aimed at addressing these challenges can begin through the identification of vulnerabilities within different migrant populations. Once identified, demographic and population-based risk analysis can reveal the extent to which mobility globalizes risks for national health systems.

### Different vulnerability levels for different migrant populations

Some health factors associated with migration are simply a function of the size of the populations on the move, and can be considered as affecting all migrants. There are specific factors associated with vulnerability, risk of illness, and adverse health outcomes that are not equally distributed across migrant groups. They may be relatively more prevalent in some migrant cohorts reflecting uneven influences of behavioral, environmental, genetic, biological,and socioeconomic determinants of health.^51^ Migrants originating from areas of poverty, those who are forcibly displaced by conflict or environmental calamity, those with limited educational and linguistic skills, and those who are dependent on their communities for protection (such as people with preexisting health conditions, unaccompanied minors, the elderly, the young,and single-parent families) are at greater risk of adverse health outcomes.^52^ At the same time, new arrivals who are subjected to legal, economic, and/or social exclusion can be very vulnerable to contracting disease resulting from poor living environments and exploitative working conditions, including lack of access to health care and preventive services.

### Globalization of health risks

Through a combination of genetic or biological, behavioral, environmental, and socioeconomic determinants of health, many global populations display major differences in the frequency and expression of poor health. The introduction of population movement into an area characterized by sustained differences in measures of population health allows for the transfer or elaboration of these characteristics between locations.^53^ This is a concept with far-reaching implications for health maintenance and promotion, disease prevention, intervention and health-services management, and education and training programs. In the sphere of infectious diseases, population mobility is one of the underlying factors in the emergence and reemergence of diseases of international public health importance as shown by communicable disease outbreaks.^54^ Prevalence disparities between migrant source and destination countries can also exist for noninfectious diseases and conditions, although the direction of disparity from high to low prevalence and vice versa may vary depending on the condition being studied. As a consequence, migrant-receiving nations are sometimes required to respond to adverse health outcomes that originate beyond their jurisdiction and normal planning considerations. In addition, migrants who are subjected to legal, social, or economic isolation and deprivation may develop levels of ill health much different from those seen in the local or host population. Taken together, evidence clearly suggests that health interventions and attempts to mitigate adverse health outcomes in migrant communities may require approaches that differ from those required by the locally born community.^55^, ^56^

### The impact of migration on national health and disease epidemiology

Many economically advantaged nations have gained the benefits of long-standing and effective public health and disease-control programs. The effective control of tuberculosis in much of the high-income world means that the major remaining public health challenges presented by the disease in developed countries are related to migration.^57^ In terms of noninfectious conditions and chronic diseases, programs directed at detection and treatment for malignancies (cervical, breast, and colon), interventions to manage anemia and some endocrine disorders, and the significance of maternal–child health issues have resulted in the moderation of the impact of these illnesses locally. Migration from less economically advantaged areas will affect the epidemiology of diseases in ‘low-incidence’ host environments.^58^ This is particularly true for diseases that occur at very low incidence levels, or which have been virtually eliminated locally.

The relationship between migration and disease epidemiology at the destination of the migrant is not always negative. Differential risk and variability in adverse health outcomes may exist between migrant and host populations for a number of diseases. Variations in prevalence may go in several directions or vary over time and across generations. Health impacts of population mobility may occur in an epidemiologically neutral context. Large populations of labor migrants moving within Asia or Western Europe represent situations in which there may be no significant health disparities between mobile and host populations, and any significant migrant effects on health instead are related to the scale of demand for services. Finally, population mobility may result in situations in which new arrivals exhibit more positive health characteristics than those observed in the host population. Examples of the latter type are observed in the context of some noninfectious or chronic diseases, such as cardiovascular diseases and eating disorders, and are described as representing the ‘healthy immigrant effect.’^59^, ^60^ Even this ‘healthy immigrant effect’ concept varies depending on time, social situation, and clinical condition.^61^

Although preexisting health conditions and illnesses can be associated with changes in disease patterns, the act of migration can also pose new threats and health risks. Postmigration public health impacts include the consequences of ill health when newly arriving migrants experience isolation, social exclusion, and/or poverty.^62^ In situations in which migrant communities’ access to health or social services is limited, post-arrival susceptibilities may increase, manifested by the expression of a more severe or advanced disease.^63^

Programs and strategies designed to promote and improve the health of migrants and mobile populations vary between nations. They often reflect national health priorities and local migration dynamics. Examples include developing migrant-friendly clinics and hospitals,^64^ ensuring that some categories of unauthorized migrants, such as those without documents, can receive care without being reported to immigration authorities,^65^ providing medical care or screening guidelines indicating the role had by mobile populations in the epidemiology of a given disease or illness,^66^ and in terms of public health risk, considering migrant and mobile populations in emergency and contingency plans for disease risk mitigation.^67^

### Health system challenges

Migration-associated adverse health outcomes present two sets of challenges to health systems in migrant-receiving nations. The first challenge is the early appreciation and recognition of the diversity and disparity components of the population, which could result in significant migrant health issues. Early recognition can be achieved through effective engagement of the health-services system by a health professional or the migrant. The size of the migrant population and the diversity within migrant communities and populations can mask significantly different groups with disease vulnerabilities. Health practices may differ significantly by group, particularly in health-promotion strategies and approaches to disease screening between source and host nations (for example, hypertension, diabetes in pregnancy, colon, uterine or breast cancer). These differences may also extend to infectious disease prevention and control measures, such as vaccination or outbreak response. If health indicators are collected or recorded in terms of broad administrative classifications, such as ‘migrant’ or ‘place of birth,’ health risks in populations composed of internally displaced, refugees, or trafficked individuals may be obscured.

The second set of challenges is related to access to care for migrant populations. Even with the recognition of adverse health outcomes in migrant populations, providing secure access to equitable health services for populations of migrants can be difficult. These difficulties can exist even in nations with long-standing immigration programs. For nations that have only recently begun to deal with the growing dynamics of international migration, the difficulties can be much greater. Professional and population education, training, and orientation to societal values in health promotion and maintenance, disease prevention, and health services utilization also take time and commitment to achieve.^68^

New and evolving populations of migrants and mobile populations can result in rapid arrival and growth of large communities with diverse characteristics, which include social, linguistic, cultural, and economic status; these can be associated with disparate health outcomes. Access to and utilization of health and medical services by some foreignborn communities may have a different pattern than those of the host population.^69^, ^70^ Specialized services encompassing linguistically and culturally competent providers, designed for the problems of migrants, will be required to ensure adequate health-care programs and service delivery models. Similar features may need to be integrated into public health and disease-control programs designed to mitigate health threats or risks.^71^ This is reflected in the need to have educational and instructional information prepared in the language of migrants at an appropriate level for comprehension, and the need for translation or visual tools to deliver messages in a culturally appropriate manner.^72^

Depending on the location and health sector capacities, these forces can affect the design and function of the health program. Migrant-specific programs may be more effective for the migrant community, but they may engender additional costs and resource demands. Additional challenges occur as a result of migrant diversity itself and can be seen in many locations where demands or needs for culturally competent health services can extend across several nationalities and ethnicities. Strategies to deal with these situations include (1) support for acculturalization and integration to allow migrants to better use domestic medical and health services and (2) the provision of migrant-specific or migrant-friendly health services.^73^ National, regional, and municipal differences in migrant history and demography make it unlikely that a single approach will be applicable to all venues.^74^ However, modern information technology and networking does provide opportunities for the sharing and exchange of best practices and information across cultural environments.^75^, ^76^

The legal or administrative status of the migrant does affect access to services and care.^77^ Migrants in an unauthorized situation and some foreign-born women^78^ have been shown to have a lower utilization of health services than the local population. For example, health services may be too costly for migrants who do not have health insurance coverage. Although linguistically appropriate health services are available and affordable, they may not be used because of migrants’ lack of information about their rights and entitlements or out of fear for deportation.^79^

### Limitations to traditional responses to the health challenges of migration

Traditional approaches to health and migration frequently deal with specific diseases, primarily communicable diseases of public health significance that may be associated with the arrival of migrants.^80^, ^81^ Coordinated attempts at the international level to manage infectious disease transmission were organized and consolidated into the International Health Regulations (IHR), which was revised in 2005.^82^

Some nations with integrated and long-standing immigration programs have systematically screened applicants for permanent residency status (immigrants) and some other classes of mobile populations (such as temporary resident applicants, including foreign students or migrant workers^83^), for various health conditions and illnesses. Immigration medical screening, quarantine, and isolation have been used in attempts to address the possible introduction of health threats by exclusion.^84^ Major immigration-receiving nations continue to use these processes to reduce the impact of health disparities in arriving mobile populations.^85^ Important as they are from a legal and administrative perspective, programs and policies that continue to embrace responses of inspection and exclusion will be increasingly costly and ineffective in the context of modern migration and population mobility.^86^ Furthermore, attempting to manage or mitigate health risks in arriving travelers, when many of the health risks may be latent or subclinical, without affecting international travel and commerce is operationally and logistically impractical.

Modern migration is an integral component of the broader process of globalization and is intimately linked to other nonhealth aspects of globalization, such as global trade and economics, safety and security, and environmental and climatic changes.^87^ Population mobility underpins the labor and economic demands for human capital. It also helps mitigate the social, demographic, and economic impacts of aging populations in many economically advanced nations where increased migration is required to sustain labor markets and population growth. In addition, modern migration is greater in magnitude and more diverse in health demographics than the traditional immigration process. At present, many people migrate temporarily or travel back and forth between their community of origin and their destination. As international migration will be an increasingly important aspect of human activity, improving the health of migrants and reducing adverse health outcomes related to migration can be expected to be a growing concern globally.^88^

Some migrant-receiving nations are beginning to appreciate the impact of chronic illnesses in migrants. This includes the demand on and the use of health and medical services by foreign-born populations, and the impact of lifestyle and behavioral factors on health and the health sector.^89^–^93^ Standardized approaches to manage health implications associated with population mobility have been proposed, which extend beyond traditional immigration administrative processes to encompass an integrated health framework for modern population mobility (see Table 3).

**Table 3:** Migration health paradigm (modified from Gushulak and MacPherson^53^)

*Factor*	*Feature*	*Implication*
1. Phases of migration	Pre-departure phase	Each phase of the migration process contributes to a carry over of the preexisting and experiential influences of that phase including any trans-generational consequences of the migration
	Transit phase	
	Arrival phase	
	Post-arrival phase	
	Return phase	
2. Prevalence gaps	Increased	Inter-regional differences in the frequency and duration of health or disease
	Neutral	
	Decreased	
3. Population health determinants	Socioeconomics	The inter-dependent factors and measurements related to well being, health, and life expectancy at birth
	Genetics/biology	
	Environmental	
	Behavioral	
4. Policies and procedures––administrative	Local	The administrative features at each level of governance and regulation that define the policies and procedures related to migration
	Regional	
	International	
Perceptions of threat/risk to health	Threat identification	The health and civil jurisdiction identification and management of the perceived threats and real risks to health, health systems, health services, and public health
	Risk assessment	
	Risk management	
	Acceptable risk	
	Residual risk	
	Managed risk	

## Modernizing strategies to manage the health challenges of migration

### Managing health threats, risks, and challenges in a global context

The need for modern and global approaches to population mobility and health is not an abstract goal. Considerable attention in the field of migrant health is devoted to the investigation and improved documentation of health indicators among migrant populations in receiving nations.^94^ However, many of the health threats, risks, and challenges related to health outcomes due to migration result from factors and influences present outside the jurisdiction and hence the direct influence, of the migrant-receiving nations.^95^, ^96^ Even in nations where long-standing immigration programs are a component of national policies, the health aspects of migration may not be addressed systematically^97^ and much of the attention toward migrant health issues is only national in scope.^98^ Some regional strategies have been proposed, but analysis suggests that they may not be evenly applied or supported.^99^, ^100^ As a result, there is a paucity of systematic programs and policies to support the health of migrants. To improve global health management and preventive health practices, there is a need for coordinated international actions and partnerships between governments and organizations in nations of origin, transit, and destination. Studies have suggested that primary health prevention endeavors such as tuberculosis control^101^ in countries of migrant origin are more economical over the longer term than traditional immigration screening programs and policies. They address better universal access in support of equity and the right to health, and have secondary preventive benefits manifested through the improvement of health indicators in migrant source countries.^102^

There is growing interest in and appreciation of efforts to address the importance of health and migration at the global level. In 2008, the WHO World Health Assembly resolved to take on the issue through its member states by adopting a resolution on migrant health.^103^ Approaching the topic through coordinated, international action will require considerable changes in many current national and regional health strategies and disease-control policies. National programs based on immigration status, nationality, historical roles of national borders, and traditional travel patterns will need to be redesigned to allow for equitable access to health services for migrants, and greater exchange of information and data to improve research into migrants’ health. Threats, risks, and challenges will need to be conceptualized in terms of mobility and population dynamics, and to consider migrant origin and transit practices.^104^

### Developing multisectoral approaches

The health of migrants is intrinsically linked to all determinants of health but particularly to the unequal distribution of socioeconomic determinants, including income status, housing and accommodation, education, nutrition, sanitation, and employment.^105^, ^106^ As a consequence, societal responses will be most effective if they are multidisciplinary and involve stakeholders from all relevant sectors working together to reduce adverse outcomes and improve the health of migrants.

International dialogue and activities in the field of migration are centered around the principles and policies of more effectively managing migration for the benefit of origin and recipient nations.^107^ Sustaining and improving the health of migrants is a lateral issue that must be integrated into all aspects of migration management.^108^ This implies integrating migration into health policies and strategies that are directly related to desired health outcomes. It implies an increasing awareness among health-care providers and educators, as well as migration policy makers on how to address health threats, risks, and challenges associated with or resulting from population mobility and disparities in health services between geographical locations.

### Integrated migration health policies

A systematic approach to managing adverse migration health outcomes must reflect and integrate the several patchwork policies that have been evolving in many nations for more than a century to deal with situational aspects of migration. At present, various policies exist that address issues related to the health of trafficked and smuggled migrants, labor migrants,^109^ those traveling for medical and religious reasons, and for those applying for formal immigration. Other broader policy instruments deal with aspects of health for *bona fide* refugees and asylum seekers or refugee claimants, detained and incarcerated migrants, and for those being returned or deported.

Integrated health policies that respect the rights of migrants will greatly facilitate coordinated approaches. These must be based on standardized international terminologies and principles reflecting the tools of the UN, international organizations, and national programs. Systematic actions that support migrant health improvement access to health services, and those that address the specific vulnerabilities of certain migrant populations, will assist nations in developing programs to meet current and future demands.^110^ These measures are in the global and national public health interest of sending and receiving communities from a social equity and equality perspective.^111^

### Prioritized programs to reduce disparities responsible for the greatest health risks

Several of the adverse health outcomes related to migration, particularly those associated with infectious diseases, are already the subject of international and in some cases global attention. Many of these diseases are being addressed through initiatives that involve international and regional programs dedicated to improving global health. They include international efforts to expand immunization (GAVI),^112^ reduce the impact of high-burden diseases (such as tuberculosis, HIV/AIDS, and malaria),^113^ manage the implications of pandemic influenza,^114^ and improve public health responses in general (IHRs).^115^ Although migration and population mobility may feature in some aspects of these endeavors, there is a paucity of integrated collective action on migration-associated components. Integrating a migration component into these activities can facilitate the global approach to disease control and demonstrate immediate benefit for both source and recipient nations. Mobile populations are one of the means by which locally arising risks can become global challenges. Mitigation programs and control strategies must encompass migration components in terms of both threat-to-risk assessment and intervention planning. The importance of these issues has been noted during responses to global health events such as SARS (2003) and the more recent pandemic influenza (2009) event, wherein travel-related control measures included screening, inspection, isolation, quarantine, and exclusion. The scale of migration and population mobility has required many of these responses to have cultural and linguistically appropriate services.

To be effective, such programs need to reflect the ongoing health impacts of migration that extend well beyond the ‘immigration’ process. A relatively new phenomenon in international population mobility is the number of migrants who, greatly facilitated by modern travel industry, return to their place of origin to visit relatives or friends; they are known as VFR (visiting friends and relatives) travelers. VFR travelers typically take longer trips, stay in local homes or accommodations, eat locally prepared meals, and take fewer pretravel precautions. Many VFR travelers return to their country of origin with children who were born at the new place of residence and lack the immunity their parents acquired before migration. These migrant-related populations of VFR travelers have been identified as being associated with increased adverse health outcomes.^116^, ^117^

### Education and training in health and migration

Processes of migration and population mobility have complex ethical, legal, administrative, and social components that relate to the health of the migrant and host communities. Studies have shown that the lack of familiarity with migrant health conditions or the nature of health determinants in migrant communities can negatively affect the effectiveness of care.^64^ Better understanding of the nature of the health aspects of migration can prevent some adverse health outcomes in international migrants through activities that support the early detection and treatment of health problems in these populations.^118^

This is accomplished through early access to and availability and affordability of health care for newly arriving migrants. Minimum standards for the provision of linguistically and culturally appropriate tools that assist in health-care delivery^119^, ^120^ are emerging health-system requirements in some nations.^121^ As the world becomes increasingly mobile, multicultural health-care providers in many locations will need to acquire greater capacity to understand, study, and address health needs of migrant communities.^122^

## Conclusions

Migration and international population mobility are facts of global life. The volume of mobile populations within and beyond national boundaries is bringing increasingly high numbers of diverse and disparate populations together. This may occur in areas where traditional administrative approaches to managing migration processes cannot address the health-care needs of migrant populations nor the impacts that these populations may have on transit, recipient, and home communities. Addressing the health needs of migrants improves migrant health, avoids stigmatization and marginalization, may reduce long-term health and social costs, protects global public health, facilitates integration, and contributes to social and economic cohesion and development.

This paper posits that many of the health challenges associated with modern migration have their origins in the international and global extension of inter-regional disparities in health indicators and determinants. Mobile populations provide population bridges across these gaps in health indicators, and effectively globalize risks and negative health outcomes.

National and international policies and programs reflecting existing geo-political boundaries represent traditional approaches to dealing with health and migration but are becoming progressively ineffective in the face of migration that is growing in volume and diversity, while extending across locations with disparate health determinants and outcomes. Related to specific migrant populations or individual diseases and varying between nations, these traditional administrative approaches may not represent the most effective methods and means of meeting the current or future needs of an increasingly diverse and globalized population. The recent resolution by the WHO World Health Assembly^103^ reflects the growing international recognition of the importance of the health effects of modern population mobility.^123^

Addressing migration health needs through a global lens will be best achieved with regional and global strategies that consider public health aspects previously considered to be limited to national needs. Policies, programs, and health services for migrants may still be prepared and delivered at a local and national level, but they should be based on and reflect international and global principles^124^ and standards. Systematic and integrated approaches to migration health, based on global processes and migrants’ rights, represent an effective method of managing these issues.
